# Changes in Task-Related Functional Connectivity across Multiple Spatial Scales Are Related to Reading Performance

**DOI:** 10.1371/journal.pone.0059204

**Published:** 2013-03-27

**Authors:** Jane X. Wang, James Bartolotti, Luis A. N. Amaral, James R. Booth

**Affiliations:** 1 Department of Chemical and Biological Engineering, Northwestern University, Evanston, Illinois, United States of America; 2 Department of Communication Sciences and Disorders, Northwestern University, Evanston, Illinois, United States of America; 3 Department of Medical Social Sciences, Northwestern University Feinberg School of Medicine, Chicago, Illinois, United States of America; 4 Northwestern Institute on Complex Systems, Northwestern University, Evanston, Illinois, United States of America; 5 Howard Hughes Medical Institute, Northwestern University, Evanston, Illinois, United States of America; National Research & Technology Council, Argentina

## Abstract

Reading requires the interaction of a distributed set of cortical areas whose distinct patterns give rise to a wide range of individual skill. However, the nature of these neural interactions and their relation to reading performance are still poorly understood. Functional connectivity analyses of fMRI data can be used to characterize the nature of interactivity of distributed brain networks, yet most previous studies have focused on connectivity during task-free (i.e., “resting state”) conditions. Here, we report new methods for assessing task-related functional connectivity using data-driven graph theoretical methods and describe how large-scale patterns of connectivity relate to individual variability in reading performance among children. We found that connectivity patterns of subjects performing a reading task could be decomposed hierarchically into multiple sub-networks, and we observed stronger long-range interaction between sub-networks in subjects with higher task accuracy. Additionally, we found a network of hub regions known to be critical to reading that displays increased short-range synchronization in higher accuracy subjects. These individual differences in task-related functional connectivity reveal that increased interaction between distant regions, coupled with selective local integration within key regions, is associated with better reading performance. Importantly, we show that task-related neuroimaging data contains far more information than usually extracted via standard univariate analyses – information that can meaningfully relate neural connectivity patterns to cognition and task.

## Introduction

The operation of the human brain depends on a complex, hierarchical system of interactions that we are now beginning to probe through the use of functional imaging tools and connectivity analyses. Reading, in particular, is a high-level function supported by a widely distributed and dynamic system of brain regions acting in concert [1], [2], and proficiency levels are highly variable even in individuals without diagnosed reading disability. Recent findings demonstrate significant differences in structural or functional connections between key reading areas in subjects with reading disability versus controls [3]–[5], but few investigations of large-scale connectivity patterns related to reading have been conducted.

Structural and functional networks of the brain have been found to display nonrandom network properties [6], which can be characterized using graph theoretical methods that quantify the statistical properties and distributions of connection patterns. Such analyses require conceptualizing brain connectivity as a network of elements (i.e. brain areas) and pairwise connections (i.e. inter-areal interactions) between them, with enough elements and connections such that statistical regularities can be detected. Graph theory allows for the ability to quantify complex network structure and to locate nodes with special network properties (see [6], [7] for comprehensive reviews of graph theoretical applications to brain connectivity). We distinguish these large-scale, graph theoretical analyses from seed- or ROI-based analyses that restrict the areas investigated to *a priori* defined anatomical regions of interest and do not necessarily investigate general network patterns in connectivity.

Prior applications of graph theoretical methods to resting-state functional connectivity data have been promising, demonstrating that brain networks can be characterized as small-world, modular, and hierarchical systems [6]–[10]. Group-level fMRI connectivity studies of the resting state have elucidated important changes in brain activation patterns over development [8], [11]–[13] or neuropathology [14]–[16], but little work has been done considering individual variation in connectivity and how these can relate to task performance. A few studies have found that functional connectivity is related to the type of task being performed [17], [18], and Sheppard et al. demonstrated increased global efficiency among participants with better auditory pitch discrimination [19]. However, in general, previous task-related functional connectivity studies have not included tasks with high cognitive load or graph theoretical analyses over a large network (>100 nodes).

At the same time, the considerable heterogeneity of brain structure and networks has led to the idea that higher-level brain functions emerge from the interaction of many regions acting in different, complementary functional roles [8], [10], [20]. Recent literature has attempted to locate regions with important function or connectivity, called “hubs,” defining them based on their degree of connectedness or other network properties [10], [21], [22]. Hubs are considered nodes with special properties, but there exists no standard definition of “node” in the functional connectivity literature. In general, they are arbitrarily specified, as single voxels or groups of voxels of certain size, or as entire anatomical regions.

This study aims to relate large-scale functional connectivity patterns to individual, real-time task performance and recover hub regions functionally important to reading. We analyzed a dataset of children performing a rhyme-judgment reading task and implemented a novel, graph theoretical connectivity analysis that was data-driven and made no assumptions of relevant spatial scales. By not imposing a global threshold or signal averaging over large anatomical regions, both of which can eliminate substantial amounts of information, we investigated the intrinsic patterns of connectivity related to reading as well as how these patterns varied in relation to task performance. Importantly, we recovered hubs at a scale that was functionally relevant to our data. These methods constitute a novel extension to current functional connectivity approaches and demonstrate how brain connectivity reflects individual differences in reading performance at multiple scales.

## Results

### Behavioral Results

**Figure 1:**
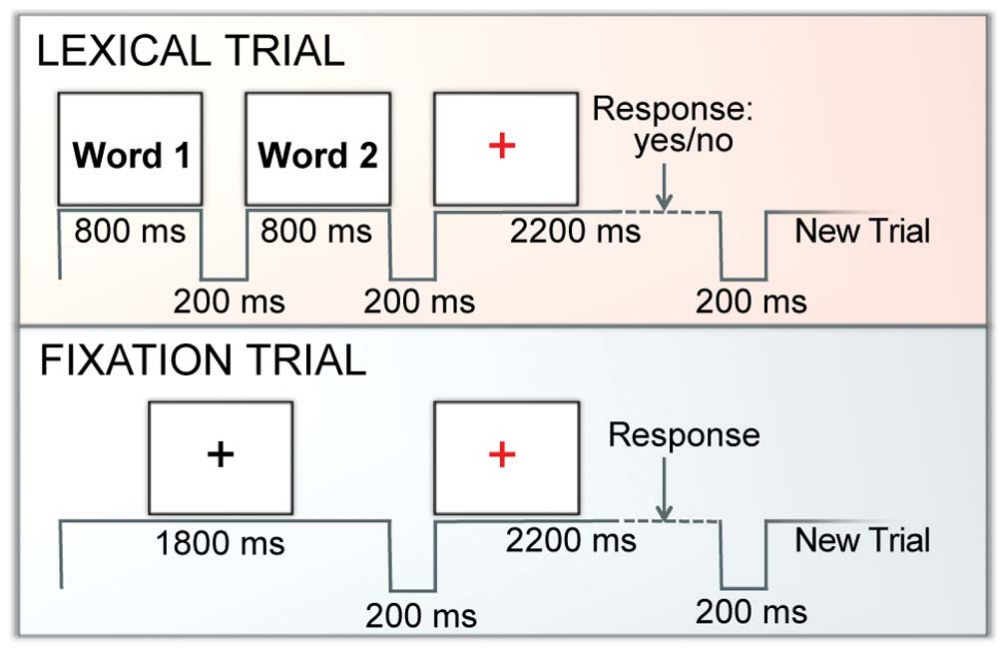
Time course of each trial and experimental design. Thirty-nine children were presented with 48 lexical trials interspersed pseudo-randomly with 36 fixation and 24 perceptual control trials in an event-related design. Each trial lasted 4200 ms, with 200 ms breaks in between, for a total of 4.4 sec each and 480 sec for the entire run. During lexical trials, words were presented sequentially for 800 ms, after which a red fixation cross appeared. The subject then had 2200 ms to provide a rhyming judgment response. During fixation trials, a black cross was displayed for 1800 ms and then was replaced by a red cross. The subject subsequently had 2200 ms to acknowledge the red cross with a button press.

Subjects performed a visual rhyme judgment task by responding to two sequentially presented words with an appropriate button press. Accuracy on these trials (Figure 1) ranged from 0.58 to 0.98, with a mean of 0.86 and a standard deviation of 0.09. The age of subjects (range 9–15 years) was marginally correlated with task accuracy (Pearson’s product moment correlation, *r*(37) = 0.32, *p* = 0.047).

### Group-averaged Connectivity Network and Constituent Subnetworks

We performed standard fMRI data preprocessing and conducted a group-level GLM analysis (see Methods for detailed description). We identified two sets of brain regions – one associated with greater activation for lexical compared to fixation trials (task-positive), and one associated with greater activation for fixation trials compared to lexical trials (task-negative). It should be noted that we applied a liberal threshold for activation (*p*<0.001 unc.), as this initial step only aimed to mask out voxels unresponsive to the task. As such, we make no claims about the significances of these activations or deactivations and simply consider these regions to be “task-responsive.” No distinctions are made between task-positive and task-negative regions for all subsequent analyses.

As expected, the task-positive and task-negative regions we identified through the GLM analysis align closely with previously known reading [23], [24] and task-negative systems [25]. The task-positive set of regions encompasses brain areas critical to reading including the fusiform gyrus and adjacent visual cortex, inferior parietal lobule, superior to middle temporal gyrus, and inferior frontal gyrus, all primarily left lateralized (see Table S1). The task-negative set of regions includes, among others, medial prefrontal cortex, posterior cingulate cortex, precuneus, medial temporal lobe, the inferior parietal/posterior temporal cortex [26] (see Table S2), areas that are known to be core regions of the default mode network, a system of brain regions found to deactivate or attenuate during attention-demanding cognitive tasks [27]–[29].

**Figure 2:**
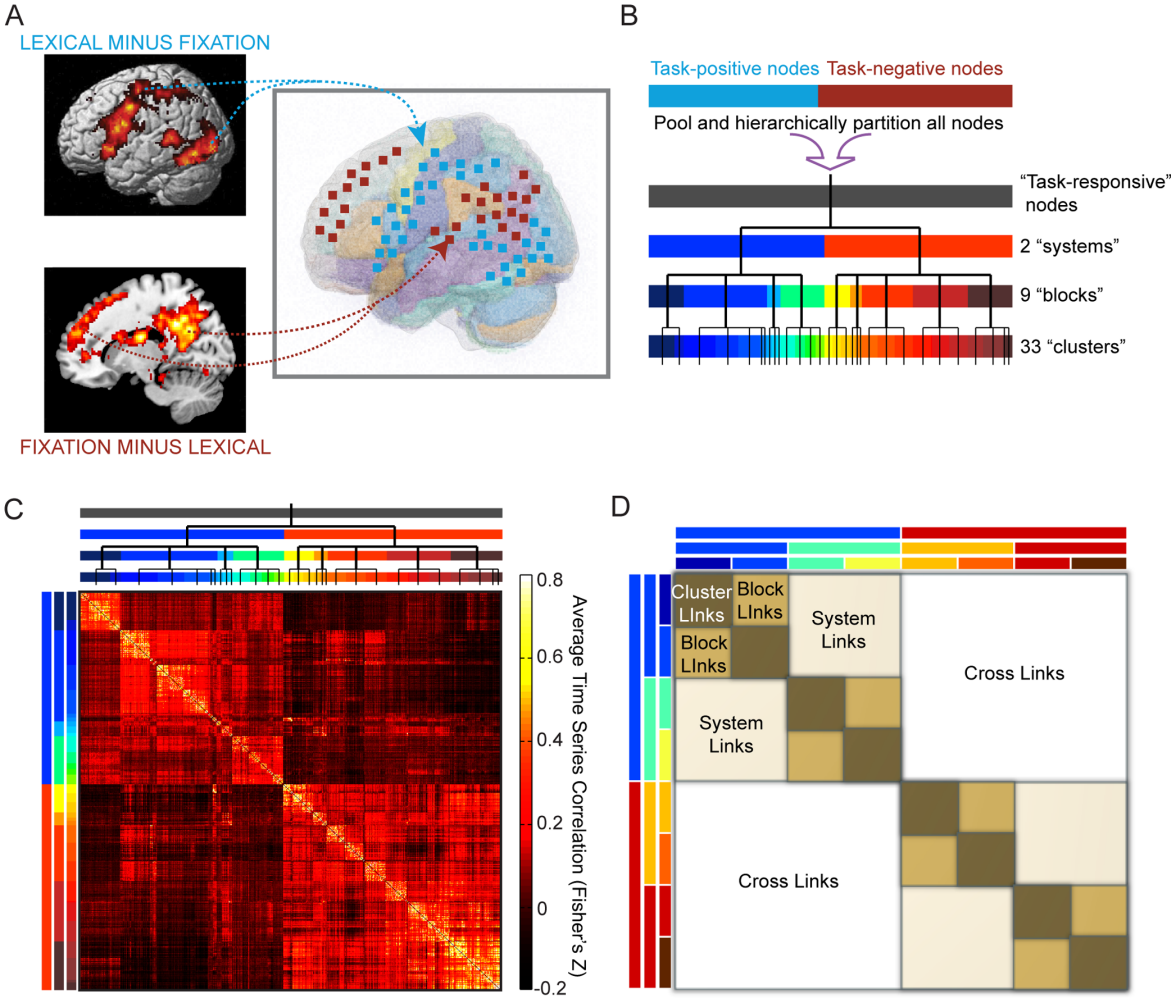
Determination of task-responsive network and hierarchical partitioning to form three levels of functional hierarchy. (A) We determine group-level GLM random effects contrasts of lexical minus fixation trials (task-positive) and fixation minus lexical trials (task-negative) to define two functional systems encompassing task-responsive brain regions. Voxels within these regions are coarse-grained to 6×6×6 mm^3^ ROIs to form the highest-resolution units of our network, called nodes. Time series of hemodynamic response are extracted from these nodes and are pairwise cross-correlated to define a weighted connectivity matrix for all subjects. (B) Schematic illustration and dendrogram of hierarchical partitioning. All nodes are pooled together and iteratively partitioned using modularity-based clustering algorithms to form a hierarchical network of relations. Each group on a higher level is partitioned to yield subgroups on the next lower level. Three levels of partitioning are defined, with various numbers of groups on each level: 2 “systems,” 9 “blocks,” and 33 “clusters.” (C) Matrix of Fisher’s Z-transformed correlation coefficients averaged over all subjects. Nodes are ordered to keep clusters, blocks, and systems contiguous. Colored bars and dendrogram along the sides indicate group membership of nodes at all three levels and correspond to those from (B). (D) Categorization of link type depends on the lowest level at which the two associated nodes are classified in the same group; i.e. same cluster (“cluster links,” brown), same block but different clusters (“block links,” tan), same system but different blocks (“system links,” beige), or between systems (“cross links,” white). Colored bars illustrate the partition of nodes into different groups at the three levels, similar to (B) and (C), but the specific groupings in (D) are conceptual only and do not represent real data.

Because we did not wish to make any assumptions about which spatial scales contained important information, we defined our highest resolution as non-overlapping, 6-mm isotropic regions of interest (ROIs) drawn from these task-responsive regions, for a total of 621 ROIs (Figure 2A–B). This particular resolution was chosen because of considerations for computational efficiency and signal-to-noise ratios, as our data were not smoothed and smaller ROIs contained much more noise. BOLD time courses extracted from these ROIs were then cross-correlated to form subject-specific connectivity matrices. These ROIs are hereafter referred to as “nodes,” and the Fisher’s Z-transform [30] of the correlation value between time series extracted from a pair of nodes is referred to as the “link weight.”

Before investigating individual differences in functional connectivity, we first characterized group-level connectivity patterns by averaging the individual connectivity matrices across all subjects. We denote the resulting matrix as the “mean connectivity strength network” (Figure 2C). In order to reveal the hierarchical organization of this network, we implemented a hierarchical clustering algorithm that is purely data-driven and partitions nodes based on modularity [31], [32] with no constraints (see Methods for details). Importantly, this algorithm handles negative as well as weighted connections. We specified three hierarchical levels of grouping (Figure 2B). The top level is referred to as the “system” level, the middle level is referred to as the “block” level, and the lowest level of groupings is referred to as the “cluster” level. A link between two nodes is labeled according to the lowest level at which the two nodes share a group (Figure 2D). Therefore, if two nodes are in separate clusters but the same block, we refer to this as a “block” link, whereas a link between two nodes in the same cluster would be termed a “cluster” link. A link between two systems (i.e. two nodes that don’t share any group) is termed a “cross” link.

The mean connectivity strength network shows that nodes within the same cluster are strongly connected to each other (via cluster links), while those more distantly related along the hierarchy – i.e., in different blocks (system links) or in different systems (cross links) – are more weakly connected. There was an overall effect for link type [Kruskal-Wallis ANOVA: *χ^2^*(3,152) = 121.53, *p*<2×10^−16^]. Cluster links (median = 0.3107) have higher weights than block links [median = 0.1971; Wilcoxon signed-rank test: *Z*(76) = 5.44, *p* = 5.26×10^−8^] which in turn have higher weights than system links [median = 0.0977; *Z*(76) = 5.44, *p* = 5.26×10^−8^]. Cross links (median = −5.20×10^−4^) have the lowest weights and are not significantly different from 0 [*Z*(38) = 0.64, *p* = 0.5209].

**Figure 3:**
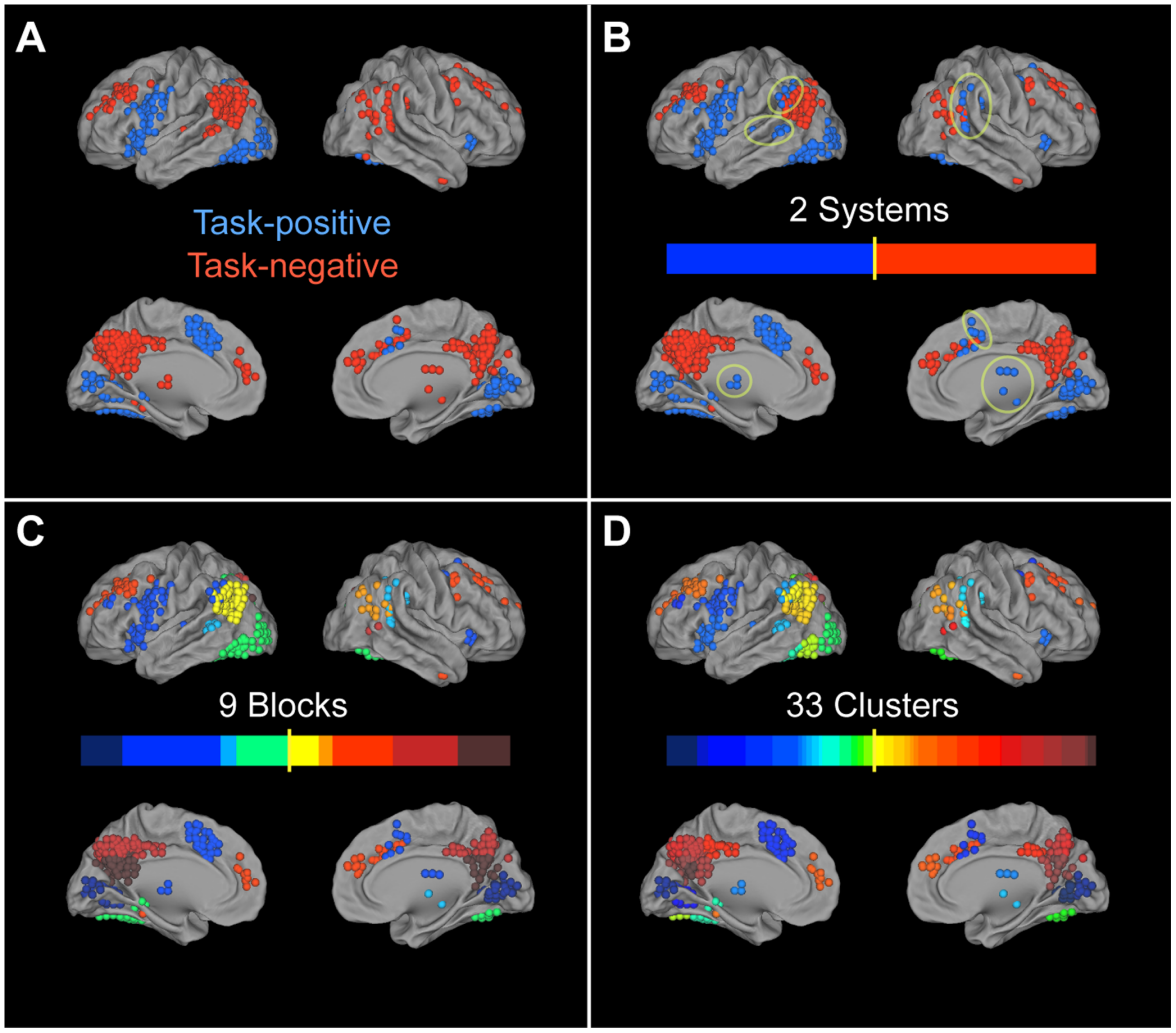
Brain maps of hierarchical partitioning. (A) Task-positive and task-negative regions defined through univariate GLM methods have high correspondence with (B) the 2 systems found through modularity-based partitioning, with the exception of a few subcortical,fronto-parietal, and temporal areas (highlighted by yellow circles). Brain maps of (C) the 9 blocks and (D) the 33 clusters show that functional groups tend to be spatially localized and confined to only a few anatomical regions.

At the top level of grouping, the nodes separate into two systems, one of which is similar to the task-positive set of regions (System 1) and one similar to the task-negative set of regions (System 2; Figure 3A–B), despite not being constrained or biased in any way by the GLM contrast. The notable exceptions are the thalamus and basal ganglia, areas of middle and superior frontal cortex, inferior parietal lobule, supplementary motor, and right middle/superior temporal cortex, all of which are classified as task-negative nodes in the GLM but are grouped with other task-positive nodes by the hierarchical clustering algorithm. At the middle hierarchical level, the nodes separate into nine blocks, four within System 1 and five within System 2 (Figure 3C). System 1 is composed of Block 1: inferior visual cortex, Block 2: an extensive set of regions spanning fronto-temporal, motor, and subcortical areas, with some nodes being task-negative (including cingulo-operculum), Block 3: a set of nodes primarily from the task-negative regions, including superior temporal and supramarginal areas, and Block 4: occipital to inferior temporal areas including fusiform gyrus. System 2 is composed of Block 5: left-lateralized posterior temporo-parietal areas, Block 6: right-lateralized counterpart to Block 5, Block 7: middle and superior frontal cortex, Block 8: a highly localized set of regions including middle cingulate cortex and superior precuneus, and Block 9: a set of regions located directly below Block 8 containing posterior cingulate cortex, cuneus, and inferior precuneus. At the lowest level, the blocks are further subdivided into 33 clusters (Figure 3D).

**Figure 4:**
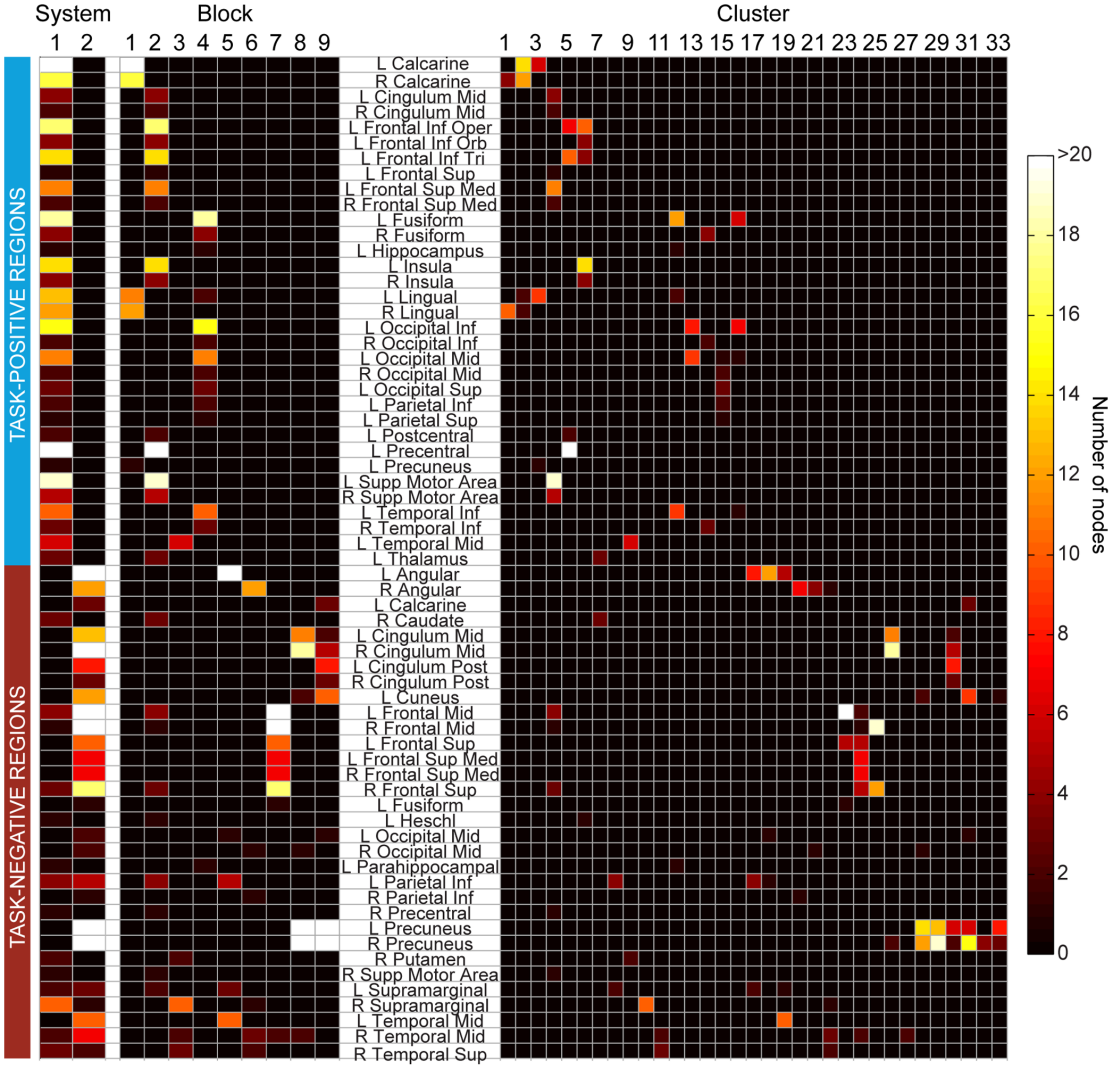
Functional groups have high concurrency with anatomical regions. Colors correspond to number of nodes belonging to the group (system, block, or cluster) as well as the anatomical region (AAL template).

Although the hierarchical clustering algorithm is purely data-driven and unconstrained by anatomical boundaries, we recovered a high concurrency with anatomical regions at the cluster level, as defined by the AAL template [33] (see Figure 4 and Tables S1 and S2). We quantified how different the data-driven cluster partition is from direct anatomical assignment by calculating the variation of information *v* [34], an information theoretic measure ranging from 0 to 1 that specifies how different two separate community structures are. We obtained *v* = 0.27, as compared to the expected *v* of 0.775±0.006 if the partitions are randomized, indicating that our clusters correspond closely to anatomical regions. Moreover, the partitions are robust (see Methods). Significantly, subsequent results do not depend on the particular partitioning used.

### Comparison of Block Partitions to Resting-state Networks

Comparison of our networks to those found in resting state literature reveals a high degree of commonality, but also interesting differences. The two systems discovered by our hierarchical partitioning algorithm are consistent with the task-positive and task-negative systems found by Fox et al. [25], but our results show more visual and less parietal activation, most likely due to task-related stimulation. Overall, System 1 appears to comprise a large subcomponent that integrates top-down control with subcortical and visual areas, while System 2 is highly consistent with previously found regions of the default network [27]. The core “nodes” of the reading network [35]–[38] – left lateralized fusiform, superior/middle temporal gyrus, inferior parietal lobule, and inferior frontal gyrus – are restricted to Blocks 2 and 4. Blocks 1 and 4 are consistent with the visual areas found by Power et al [9], and Block 2 appears to be a combination of the cingulo-operculum/somatosensory and fronto-parietal subgraphs. Block 3 encompasses the temporal portion of the default network, an interesting finding that seems to indicate that in the face of task demands the default network will reorganize into dynamic subcomponents, some of which interact with task-positive regions.

### Connectivity Patterns Correlated with Increased Performance

While the mean connectivity strength network reveals the group-averaged multi-scale organization, it hides significant between-subject differences. Most previous functional connectivity analyses ignored the signed and weighted nature of link correlations and instead applied a global threshold to yield a binary connectivity matrix, thus discarding possibly important information. Here, we incorporated the full information available in the weighted connectivity matrix and took advantage of inter-subject variability in order to elucidate how differences in connection strength reflect differences in performance at multiple hierarchical levels.

**Figure 5:**
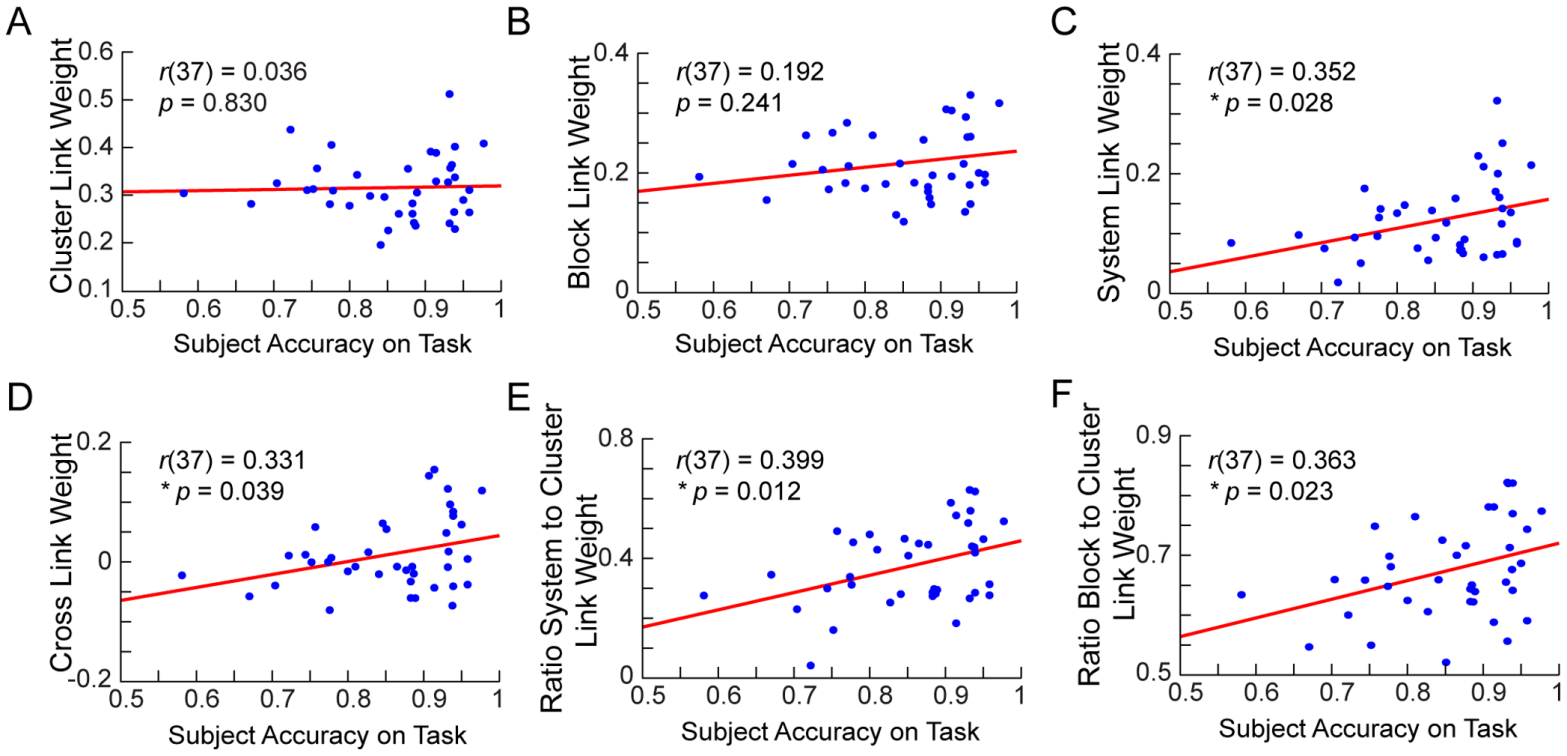
Links spanning higher levels of the hierarchy are more correlated with task accuracy. Plots of average link weight (Fisher’s Z transformed) versus task accuracy for (A) cluster links, (B) block links, (C) system links, and (D) cross links. Plots of the ratio of the average weight of (E) system links to cluster links, and (F) block links to cluster links.

For each link type (cluster, block, system, or cross), we analyzed how individual differences in connection strength are correlated to individual differences in subjects’ accuracy on the reading task. We conducted a between-subjects analysis by averaging all link weights of the same link type in each subject and then found the cross-correlation of these link weight averages with subject accuracy. Notably, we found that correlation between link weight and task accuracy is higher for nodes that are more distant along the hierarchy. As shown in Figure 5, cluster and block link weights do not significantly correlate with accuracy [Pearson’s product moment correlation; cluster links: *r*(37) = 0.036, *p* = 0.830; block links: *r*(37) = 0.192, *p* = 0.241]. However, the system link weights are significantly correlated with task accuracy [*r*(37) = 0.352, *p* = 0.028], as are cross link weights [*r*(37) = 0.331, *p* = 0.039]. In addition, the ratio of a single subject’s average system link weight to their average cluster link weight is significantly correlated with accuracy [*r*(37) = 0.399, *p* = 0.012], as is the ratio of block link weight to cluster link weight [*r*(37) = 0.363, *p* = 0.023].

### Hub Regions of the Task Network

The brain is well-known for being highly heterogeneous, in terms of both connectivity and anatomical structure. In order to characterize heterogeneity among the functional groups, we investigated hub-like properties of our clusters, rather than individual nodes, allowing our data to functionally define relevant spatial scales. We chose the clusters found at the third level of our hierarchical network as candidate hubs for the following reasons: 1) clusters were recovered through data-driven means and thus are more likely to reflect the relevant scales of our data, 2) unlike blocks or systems, clusters do not span many anatomical regions, thus making them readily interpretable, and 3) the relatively high number of clusters in our network allows for the application of graph theoretical measures, whereas such measures are not meaningful if applied to fewer numbers of elements.

**Figure 6:**
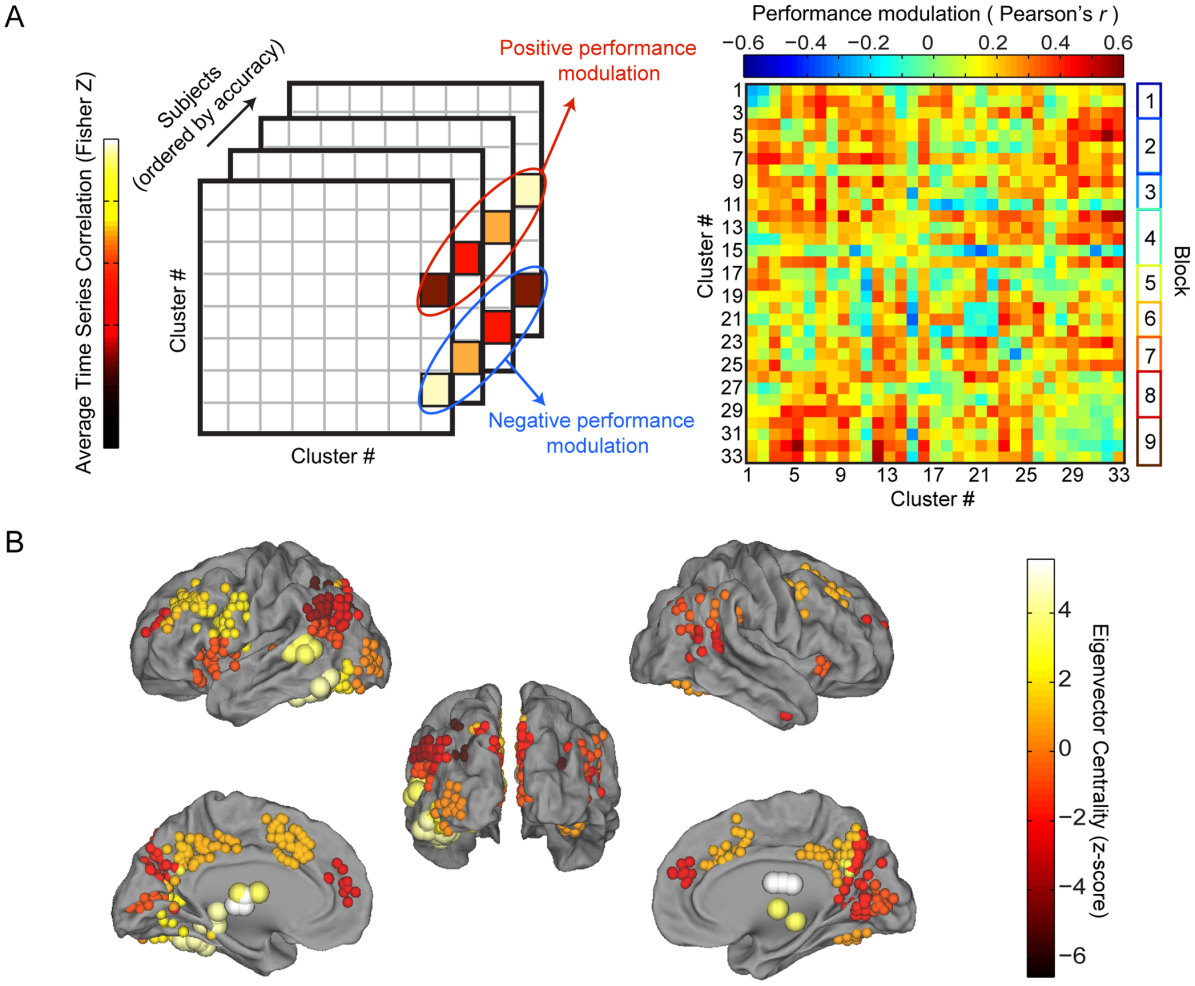
Performance modulation network quantifies modulation of correlations by task accuracy and reveals key hub regions. (A) We investigated the hub-like nature of the 33 clusters by averaging together link weights of all links within clusters as well as links between pairs of clusters to create cluster-level connectivity matrices for all subjects. Performance modulation for every link was then found by calculating the correlation of link weight with task accuracy across participants to create the performance modulation matrix. (B) We classify as hubs the clusters with the highest eigenvector centrality values calculated on the performance modulation matrix from (A). The hubs found (large spheres) encompass key reading areas such as fusiform gyrus and left temporal gyrus, as well as dorsal striatum, thalamus, and left hippocampus. Views depicted are lateral (top), posterior (center), and medial (bottom).

We located hubs based on their pattern of network embeddedness in the context of task performance. Our analysis was restricted to the performance modulation network, which quantified how strongly average link weight between clusters correlated with task accuracy (see Figure 6A–B and Methods). Conceptually, being a hub in the performance modulation network indicates that a cluster is part of a group of clusters that all display higher connectivity weights to each other as accuracy increases. As such, it is a measure defined only in relation to other elements and is therefore not a property of an individual element in isolation. Our results indicate that Clusters 12, 7, and 9 can be classified as hubs, with *z*-scores of 5.58, 5.19, and 4.08, respectively. As illustrated in Figure 6C, Cluster 12 is composed of left inferior temporal gyrus, left fusiform gyrus, and left hippocampus; Cluster 7 left thalamus and right caudate; and Cluster 9 left middle temporal gyrus and right putamen. Possibly this indicates that reading accuracy is supported by task-dependent interactivity between dorsal striatum and the core left-hemisphere regions known to be critical for reading (for related findings, see references [5], [23], [35], [38]). Hubs have marginally significant increased integration as accuracy increased [*r*(37) = 0.301, *p* = 0.055], whereas non-hub clusters displayed no integration with accuracy [*r*(37) = 0.040, *p* = 0.809].

## Discussion

The application in recent years of graph theoretical approaches to functional connectivity analyses has highlighted the complex, non-random nature of neural connectivity and interaction dynamics within the brain [32]. The majority of these analyses have been performed on the resting state, a paradigm that offers many advantages including its relative ease of collection. However, resting-state connectivity highlights commonalities across a population and probes neural interactions in the absence of any task, and thus is difficult to relate to task-related experimental results. Our study constitutes a novel extension that builds on both univariate task-related methods as well as previously implemented resting state functional connectivity methods to yield important insight into how reading, a high-level cognitive skill, relates to patterns of neural interaction.

Activation levels found through univariate analyses are not trivially related to connectivity during task performance (see Figure S1), indicating that considerable additional information can be gained by analyzing task-related neuroimaging data with connectivity methods. These results demonstrate that, during reading, the task-responsive system decomposes – based on strengths of interaction between nodes – into a hierarchical network with multiple functional scales. We found that, surprisingly, some task-negative regions are highly interactive with task-positive regions, which runs counter to expectations from univariate, GLM-based approaches and demonstrates the power of combining connectivity with task-based approaches.

We observed higher link weights in the subject-averaged mean connectivity matrix for more local links (cluster link weights>block link weights>system link weights, see Figure 2), but the opposite trend when examining connectivity across subjects and correlations with accuracy. A possible explanation is that group-averaged functional connectivity aligns with core anatomical structure [39], which tends to be stronger locally, whereas functional connections that vary across subjects are dynamic, task-specific, and involved in information transfer between anatomical regions. Our results are consistent with recent resting-state functional connectivity studies reporting general integration (increased long-range interactions) in the default mode network over the course of development [40], [41], perhaps suggesting that long-range integration is indicative of a more efficient functional brain network state which requires maturation. We extended these findings by showing that, in addition to spatial distance, the functional scale at which two regions are connected determines their connectivity and how their interaction varies with task behavior. Interestingly, cross link weights, which mostly connect task-positive and task-negative nodes, display long-range integration with task-accuracy, implicating a more distributed and complex brain network supporting fluent reading than previously thought. For instance, long-range frontal to temporal increases in connectivity could support higher levels of cognitive control, consistent with prior findings of increased long-range connectivity between these areas in adults versus children [40], [42]. The complex dependence of connection strength on behavior can only be uncovered by examining connectivity in the context of variation in task performance.

We found considerable heterogeneity among different clusters, suggesting that, despite occupying the same functional level, clusters play different functional roles in support of reading. Previous literature suggests that those with reading disability have altered activation and connectivity in the “core” reading network [4], [5], [43]: left fusiform gyrus, superior/middle temporal lobe, inferior frontal gyrus, and inferior parietal lobule. The hubs we recovered are consistent with several of these regions, but also include subcortical areas such as thalamus, basal ganglia, and hippocampus, indicating the recruitment of a wide range of brain functions during reading. Although our subjects were all healthy children, it is likely that readers with lower accuracy exhibit mild versions of the connectivity disruptions seen in children diagnosed with reading disability.

We observed that the network of hubs is more synchronized within clusters as well as between clusters in support of better reading, supporting the hypothesis that these areas coordinate separate sources of bottom-up or top-down information and that this coordination facilitates correctly performing reading tasks. No hubs were found in the task-negative regions, suggesting that, in the presence of a cognitively complex task such as reading, local integration within the default network is not helpful for performance. Collectively, these results constitute a possible neural signature for better reading in children.

There are two key advantages of our approach. First, examining subject differences in connectivity rather than group averages allows for the ability to control for artifacts that could be introduced by various preprocessing steps, such as smoothing (higher local correlations), resampling (higher local correlations), and global signal regression (introduction of negative weights). Because these steps are uniformly applied to all subjects, they should not affect between-subject differences in connectivity. A second important issue regarding connectivity analyses is how to properly define a node [9]. We argue that no assumptions of spatial scale should be made; therefore, our time series were extracted from ROIs of the minimum size that was computationally feasible and contained adequate signal-to-noise ratios. Further, brain networks contain multiple functional scales, so we allowed these scales to arise from data-driven means through hierarchical partitioning on the naturally occurring, weighted, and signed interactions.

It is important to note some potential limitations of the current study. First, because our time course consists of task, fixation, and perceptual control trials, the connectivity patterns found cannot be specifically linked to any one trial type or to the overall effect of task. Specifically, it is possible that task-related connectivity found here is partially due to either simultaneous activation of two regions from common sensory input or to underlying anatomical connectivity. However, our major results are built on differences in connectivity across subjects performing the same task and therefore cannot be an artifact of common sensory input. It is true, however, that we are unable to distinguish between anatomical connectivity and functional connectivity, and therefore care must be taken with the interpretation. Nevertheless, we would like to point out that this issue is not limited to task-related connectivity and extends to the resting state as well. Further, the mixing of trial types might contribute to the subtlety of our findings; i.e. block or system links only correlate with behavior at a correlation coefficient of *r* = 0.3–0.35. However, any such dilution of the signal would only serve to reduce our statistical power, and trials were pseudo-randomized with both fixation and perceptual control trials to reduce any systematic contaminations between trial types. Thus, our results are conservative and likely to be strengthened in a task-related block design of only reading tasks.

Second, as with any functional connectivity analysis, the results can be sensitive to preprocessing steps such as smoothing or to subject movement. Therefore, we made efforts to ameliorate or control for these effects by not smoothing our data, ensuring that ROI’s are not overlapping, ignoring links between ROIs less than 10 mm apart, and removing from the analysis all volumes with frame displacement of more than 0.3 mm.

Finally, hierarchical partitioning of the mean connectivity matrix and calculating eigenvector centrality of the cluster-level performance modulation network are analyses necessarily conducted on a measure already defined across subjects. As such, we could not define between-subject statistics for these calculations. We have made efforts to quantify the robustness of our findings by conducting various robustness tests and showing that leaving out multiple random subjects did not significantly alter the results of these analyses.

Recently, Gonzalez-Castillo et al. [44] have pointed out that a wealth of information is ignored when only considering highly significant activation maps. Consistent with this, our results demonstrate that the neural substrates of cognition entail interactions and dynamics that can’t be entirely captured by univariate, task-related methods. Connectivity analysis offers insight into data that is complementary to univariate analyses, and integration between these two sources of information is crucial for forming deeper understanding of higher-level cognition such as reading. Tying these results with the resting state literature will also prove vital to answering intriguing questions such as: How does connectivity within the “default” network change in support of various types of tasks and across different conditions? Many open questions remain regarding the relationship between functional connectivity and univariate studies, including the exact extent of their mutual information. The methods presented here could pave the way for understanding such questions and at the same time provide a cost-efficient, quick method of analyzing previously acquired task-related data, thus revealing promising avenues of future research.

## Materials and Methods

### Ethics Statement

Subjects, as well as parents or guardians, gave written, informed consent to participate in this study. The study protocol was approved by the Institutional Review Board at Northwestern University.

### Subjects

Thirty-nine healthy children recruited from the Chicago metropolitan area participated in the study (21 females, mean age = 11.92, range = 9–15). All children were native English speakers with normal hearing and normal or corrected-to-normal vision, were free of neurological diseases or psychiatric disorders, and were not taking medication affecting the central nervous system. Parents of children were interviewed to ensure that children did not have a history of deficits in reading, language, or attention. Children were right-handed (mean = 80, range 55–90) according to the 9-item Likert-scale questionnaire (−90 to 90, positive scores indicate right-hand dominance). Children were given two standardized intelligence tests: Wechsler Abbreviated Scale of Intelligence (WASI) [45] and the Word Identification test from the Woodcock Johnson Tests of Achievement (WJ-III) [46]. All subjects included scored within 2 standard deviations of the average score (WASI: 111.8±15.5; WJ-III: 108.2±11.0).

### Task and Experimental Design

Data was collected in a pseudo-randomized event-related design, the initial results of which were published previously [47]. The experiment consisted of two consecutive runs of 108 trials, including 48 lexical trials, 36 fixation trials, and 24 perceptual baseline trials. However, only the first run was analyzed in each subject because of concern over habituation and practice effects as well as fatigue, especially since this study was conducted with children. In the lexical trials, two words were presented visually in a sequential order and the participant had to determine whether the words rhymed. Each word was presented for 800 ms followed by a 200 ms blank interval. A red fixation-cross appeared on the screen after the second word, indicating the need to make a response during the subsequent 2400 ms interval. Twelve word pairs were presented in each one of four lexical conditions to give a total of 48 lexical trials that independently manipulated the orthographic and phonological similarity between words. In the two non-conflicting conditions, the two words were either similar in both orthography and phonology (O+P+, e.g. *dime-lime*), or different in both orthography and phonology (O−P−, e.g. *press-list*). In the two conflicting conditions, the two words had either similar orthography but different phonology (O+P−, e.g. *pint-mint*) or different orthography but similar phonology (O−P+, e.g. *jazz-has*). Participants were instructed to press a button with their index finger for rhyming word pairs and to press a different button with their middle finger for nonrhyming word pairs. Conflicting and non-conflicting pairs were employed so that participants could not make rhyming decisions on spelling alone. All words were monosyllabic words (4–7 letters), and were matched across conditions for written word frequency in children by using The Educator’s Word Frequency Guide [48] and for written and spoken word frequency in adults by using CELEX [49].

Thirty-six fixation trials were included as a resting baseline. In the fixation condition, a black fixation cross was presented for the same stimulus duration as the lexical stimuli and a button was to be pressed with the index finger when the black fixation-cross turned red. It should be noted that our fixation trials consist of a motor response in order to control for motor movement during task trials. In addition, 24 trials in two control conditions were used as a perceptual baseline for a related study (see [47], [50] for detailed methods). For these conditions, participants were visually presented with pairs of either single symbols or symbol strings with the same timing as the lexical trials and had to determine if they matched. The lexical trials were interspersed with the fixation and control trials in pseudo-random fashion to minimize the effect of contamination from previous trials, as recommended by Burock et al [51], for a total of 108 trials. The order of stimuli within the task was fixed for all subjects. All trials lasted for a total of 4.4 seconds.

After informed consent was obtained and the standardized tests were administered, participants performed one run of the experimental task in a custom simulator scanner in order to ensure their understanding of the tasks and to acclimatize themselves to the scanner environment. In addition, participants were trained to minimize head movement in the simulator scanner using online feedback from an infrared tracking device (Flock of Birds, Ascension Technology Corporation, Milton, VT). Different stimuli were used in the practice and in the scanning sessions. Scanning took place one week after the practice session. Accuracy of performance in the scanner and reaction times from the onset of the second item in each trial were recorded.

### MRI Data Acquisition and Preprocessing

Images were acquired using a 1.5-T GE (General Electric) scanner, using a standard head coil. Head movement was minimized using vacuum pillow (Bionix, Toledo, OH). The stimuli were projected onto a screen and viewed through a mirror attached to the inside of the head coil. Participants’ responses were recorded using an optical response box (Current Designs, Philadelphia, PA). The BOLD (blood oxygen level dependent) functional images were acquired using the echo planar imaging method. Image orientation was oblique axial. The following parameters were used for scanning: TE = 35 ms, flip angle = 90°, matrix size = 64×64, field of view = 24 cm, slice thickness = 5 mm, number of slices = 24; TR = 2000 ms. One run contained 240 time points and lasted 480 seconds. In addition, structural T1-weighted 3D image were acquired (SPGR, TR = 21 ms, TE = 8 ms, flip angle = 20°, matrix size = 256×256, field of view = 22 cm, slice thickness = 1 mm, number of slices = 124), with the same orientation as the functional images. All images were acquired in the axial plane starting at the top of the brain, so coverage of the cerebellum was not complete for either the structural or functional scans.

The functional and structural data were preprocessed within each run by using SPM8 (Wellcome Trust Centre for Neuroimaging, http://www.fil.ion.ucl.ac.uk/spm). The functional images were spatially realigned to the first volume to correct for head movements using second degree b-spline interpolation. Functional volumes were slice-time corrected to the first slice and sinc interpolation was used to minimize timing errors between slices. The functional images were co-registered with the corresponding structural MRI using mutual information optimization and normalized to the MNI (Montreal Neurological Institute) template, with voxel size 2×2×2 mm^3^. Data were not smoothed in order to minimize spurious correlations at small distances. The first four volumes of each participant’s functional data, which contain transient T1 effects, were discarded from statistical analysis.

### Defining Functional Networks

The 4 lexical, 2 control, and fixation conditions were modeled as conditions of interest. All conditions were treated as individual events and modeled with a canonical hemodynamic response function. Six-parameter rigid body movement was included as a nuisance regressor. A group-level, random-effects analysis [52] identified brain areas most significantly active for the lexical>fixation conditions and for fixation>lexical conditions. Significance was determined by resampling to 6×6×6 mm^3^ cells and taking only cells that were significant at p<0.001, uncorrected, using a two-sample *t*-test in SPM. Cells significant in the lexical>fixation contrast (262) were denoted “task-positive” nodes, signifying brain areas most associated with experimental task-related activation. Cells significant in the fixation>lexical contrast (359) were denoted “task-negative” nodes, signifying areas most associated with task-related deactivation [25].

This particular resolution of 6 mm isotropic “nodes” was chosen in order to create relatively small, uniformly sized ROIs that avoid any assumptions of spatial scale. This presents a computationally efficient method that helps to stabilize the signal against noise without implementing Gaussian smoothing, which introduces spurious correlations by averaging over overlapped nodes.

### Constructing Functional Connectivity Matrices

Power et al. and Van Dijk et al. [53], [54] have demonstrated that even after motion regression, volumes with high frame displacement (FD) can introduce spurious correlations. They recommend a procedure called “motion scrubbing” to eliminate high-movement volumes prior to connectivity analyses. Before conducting motion-scrubbing procedures on our data, subjects had an average FD of 0.18 mm, collapsed across all time points and subjects. All volumes with FD>0.3 mm were eliminated and ignored in subsequent analyses. On average, 30 volumes (12.5% of all data) per subject were eliminated via this procedure, with all subjects retaining at least 50% of all volumes. The results remain largely unchanged after motion scrubbing, but all results presented include this step.

Time series of BOLD activation from each 6×6×6 mm^3^ node were calculated by averaging all voxel time series within the node, conducting motion scrubbing, subtracting the global signal, linear detrending, and filtering at 0.008–0.1 Hz in accordance with previous functional connectivity techniques. It should be noted that the motion scrubbing and frequency-filtering steps were only conducted for the connectivity analyses, and not for the univariate GLM analysis used to select our ROIs. These steps were taken in addition to the standard 6-parameter rigid body motion regression.

We then calculated single-subject connectivity matrices by pairwise cross-correlating, using Pearson’s product moment correlation, all BOLD response time series and obtaining the Fisher’s Z-transform of the cross-correlation values [30]. In the following, we denote the Fisher’s Z-transform of the correlation value between a pair of nodes as the *link weight*. This resulted in a 621×621 element matrix, where the *ij*-th element corresponds to the cross-correlation between the time series from the *i*-th node and that from the *j*-th node. As an additional measure against movement-related artifacts, we ignored links between nodes less than 10 mm apart in all analyses. The adjacency matrix of the *mean connectivity strength network* was constructed by averaging the Fisher’s Z-transformed correlation values across subjects. The Fisher’s Z transformation was performed in order to stabilize the variance for correlation values. High values in mean connectivity strength indicate a consistent (across subjects) synchronization in time dynamics between two nodes. All connection weights, positive and negative, were retained. Although currently there is no consensus on how to interpret negative connectivity weights, our analyses primarily consider *changes* in weights with reading performance rather than absolute values. As such, our results are insensitive to the actual sign of the connectivity weights.

### Dissociation between High Activation Levels and Correlation of Time Series

An important concern with conducting functional connectivity analyses on task-related data is the possibility that the correlations found between brain regions are solely driven by the magnitude of task-related activations in those regions. To address this concern, we investigated whether the connectivity between two ROIs is significantly related to the main effect of task in those ROIs. An analysis of the most activated regions (those with *t*>4) in response to lexical task minus fixation trials shows that the BOLD time series are not highly correlated with each other on average (mean *r* = 0.139±0.144; see Figure S1), as would be expected if correlation is solely driven by magnitude of activation. There are two possible explanations for this result: 1) neuroimaging data contains sufficient noise that regions with relatively high activation levels still contain enough deviations to attenuate correlations with other highly activated regions, or 2) typically, there are many variables of interest that could influence activation levels, and thus high activation with respect to one variable does not completely determine the time series (i.e. there are many possible time series that would all achieve a high *t*-value). The dissociation between activation levels and correlation in time series that we observe here suggests that task-related connectivity is not redundant with activation levels found in univariate GLM analyses.

### Statistics and Significance Testing

Connection strengths of various link types are tested by first averaging Fisher’s Z-transformed correlation values across all links at each hierarchical level, followed by significance testing using the nonparametric Wilcoxon signed-rank test, the Wilcoxon rank-sum test, and the Kruskal-Wallis ANOVA test because no assumptions of normality were made. Correlations with task accuracy are conducted using Pearson’s product moment correlation. Task accuracy is defined as the percentage correct on all lexical trials.

### Hierarchical Clustering and Partitioning

To hierarchically partition our nodes, we use a standard algorithm included in the Brain Connectivity Toolbox (Rubinov and Sporns, 2010), modified so that it will implement iterative clustering to yield a hierarchical network. We have chosen this algorithm because, to our knowledge, it is the only standard, public algorithm that works with weighted, signed networks. It implements an undirected, signed version of the Louvain method [55], a highly efficient community detection algorithm using Newman’s modularity statistic [31] as a metric. We modified this algorithm to operate iteratively to create multiple levels of partitions, so that the partitions found at one level are then themselves partitioned to create the groups of next lower level. Specifically, all task-responsive nodes are first partitioned to recover groups we call Systems, which are then partitioned to recover groups we call Blocks, and these are subsequently partitioned to recover groups called Clusters.

In order to test the robustness of our partitions, we conducted three analyses: 1) calculating partitions on the mean connectivity strength network 100 times with different random seeds, 2) calculating partitions on individual subject correlation matrices, and 3) calculating partitions on a mean connectivity strength network constructed with 3 random participants left out of the analysis, repeated 100 times. For each analysis, we can calculate the difference between all pairs of partitions by calculating the variation of information *v* [34], an information theoretic measure ranging from 0 to 1 that quantifies the amount of information lost when changing between two partitions (Brain Connectivity Toolbox, [56]). We found that the average *v* for analysis 1 is 0.016/0.036/0.086 (top/middle/lowest level; 4,450 comparisons), for analysis 2 is 0.015/0.119/0.186 (741 comparisons), and for analysis 3 is 0.028/0.067/0.105 (4,450 comparisons). We then constructed a null model, where partitions are randomized but the total numbers of clusters and the number of nodes per cluster are fixed. The expected variation of information *v* was found to be 0.311, 0.7077, and 0.7876 for the top, middle, and lowest level, respectively. Therefore, the partitions we recovered lost almost no information at the top level, little information at the middle level, and a small but nontrivial amount of information at the bottom level, although much smaller than would be expected by random chance. Notably, subsequent results do not depend on the particular partitioning used.

### Identifying Hub Regions

In order to ascertain if certain clusters are more strongly embedded than others as reading performance increases, we calculate the eigenvector centrality z-scores [57], [58] of all 33 clusters.

We first calculate average individual-subject link weights between all pairs of clusters, to obtain a 33×33×39 matrix that defines how clusters are related in each subject. The correlation coefficient between link weight and subject task accuracy is calculated across subjects for each pair of clusters to yield a 33×33 matrix of cluster-level performance modulation (see Figure 6). High values in performance modulation for a link signify increases in connection strength between two clusters for individuals with higher accuracy. We then calculate the eigenvector centrality of each cluster based on this performance modulation network and identify those with statistically significant values of eigenvector centrality as “hubs.”

Eigenvector centrality calculates how well an element is connected within the network by looking at the quality of its neighbor’s connections [10], [57], [58]. The advantages of eigenvector centrality are that it generalizes well to weighted networks and it takes higher order connections into account when assessing importance, i.e. connection to important nodes confers more importance than connection to unimportant nodes. We compute eigenvector centrality using a standard iterative algorithm defined by Newman [59]. Because eigenvector centrality is only defined for positive weights – variations which take into account negative weights exist, but do not necessarily converge to unique or stable solutions [57], [60] – we add a constant to all connection strengths.

Significance is determined by creating a sample null distribution of 1000 matrices with shuffled rows and columns and calculating centrality of all clusters. We consider a cluster to have a significant eigenvector centrality if the corresponding *z*-score (compared to the null distribution) is significant at *p*<0.01, corrected for multiple comparisons.

A leave-3-out robustness test, in which 3 random participants are left out of the analysis, indicates that the hubs recovered are highly stable. Out of 100 trials, clusters 7, 9, and 12 were assigned hub status 99, 83, and 98 times, respectively. No other clusters were comparable, with the closest being clusters 5 and 23, which were assigned hub status 12 times each. We are therefore reasonably confident that the hubs found are typical and representative.

### Visualization

All brain surface visualizations were constructed using Caret software [61] and the PALS atlas [62].

## Supporting Information

Figure S1 **High activation levels are not related to high time-series correlation.** (A) Two example time series of activation from two separate ROIs (light gray and dark gray solid lines) are superimposed on stimulus onsets for lexical (dashed green line) and fixation (dashed orange line) trials, convolved with a canonical hemodynamic response. The fixation trial stimulus onsets are inverted (onset corresponds with downward inflection) for visual clarity. Both ROIs exhibit high activation levels (*t*>6.5 for lexical minus fixation contrast), yet have very low correlation between them (Pearson’s product moment *r* = 0.085). (B) Time series from all nodes with high activation (*t>*4) are binned according to activation level, and all time series in the same bin are cross-correlated. The resultant correlations are binned along the y-axis to yield a 2-D histogram of how correlations are distributed for every *t*-value bin.
(TIF)

Table S1 **Details of anatomical regions in the task-positive areas.** Columns refer to the number of nodes within each anatomical region (as defined by the AAL atlas), spatial coordinates of the center of mass, and Brodmann Area (BA). X, Y, and Z refer to MNI coordinates. Region names: CAL: Calcarine, MCG: Middle Cingulate, IFGoperc: Inferior Frontal Gyrus pars opercularis, ORBinf: Inferior Frontal Gyrus pars orbitalis, IFGtriang: Inferior Frontal Gyrus pars triangularis, SFG: Superior Frontal Gyrus, SFGmed: Medial Superior Frontal Gyrus, FFG: Fusiform, HIP: Hippocampus, INS: Insula, LING: Lingual Gyrus, IOG: Inferior Occipital Gyrus, MOG: Middle Occipital Gyrus, SOG: Superior Occipital Gyrus, IPL: Inferior Parietal Lobule, SPG: Superior Parietal Gyrus, PoCG: Postcentral Gyrus, PreCG: Precentral Gyrus, PCUN: Precuneus, SMA: Superior Motor Area, ITG: Inferior Temporal Gyrus, MTG: Middle Temporal Gyrus, THA: Thalamus.
(DOCX)

Table S2 **Details of anatomical regions in the task-negative areas.** Columns refer to the number of nodes within each anatomical region (as defined by the AAL atlas), spatial coordinates of the center of mass, and Brodmann Area (BA). X, Y, and Z refer to MNI coordinates. Region names: ANG: Angular Gyrus, CAL: Calcarine, CAU: Caudate, MCG: Middle Cingulate, PCG: Posterior Cingulate, CUN: Cuneus, MFG: Middle Frontal Gyrus, SFG: Superior Frontal Gyrus, SFGmed: Medial Superior Frontal Gyrus, FFG: Fusiform, HES: Heschel’s Gyrus, MOG: Middle Occipital Gyrus, PHG: Parahippocampal Gyrus, IPL: Inferior Parietal Lobule, PreCG: Precentral Gyrus, PCUN: Precuneus, PUT: Putamen, SMA: Superior Motor Area, SMG: Supramarginal Gyrus, MTG: Middle Temporal Gyrus, STG: Superior Temporal Gyrus.
(DOCX)
