# Supplementary figures and images for: Changes in Task-Related Functional Connectivity across Multiple Spatial Scales Are Related to Reading Performance
Source: PLoS One. 2013 Mar 27;8(3):e59204. doi: 10.1371/journal.pone.0059204 (PMC3609744; doi:10.1371/journal.pone.0059204)

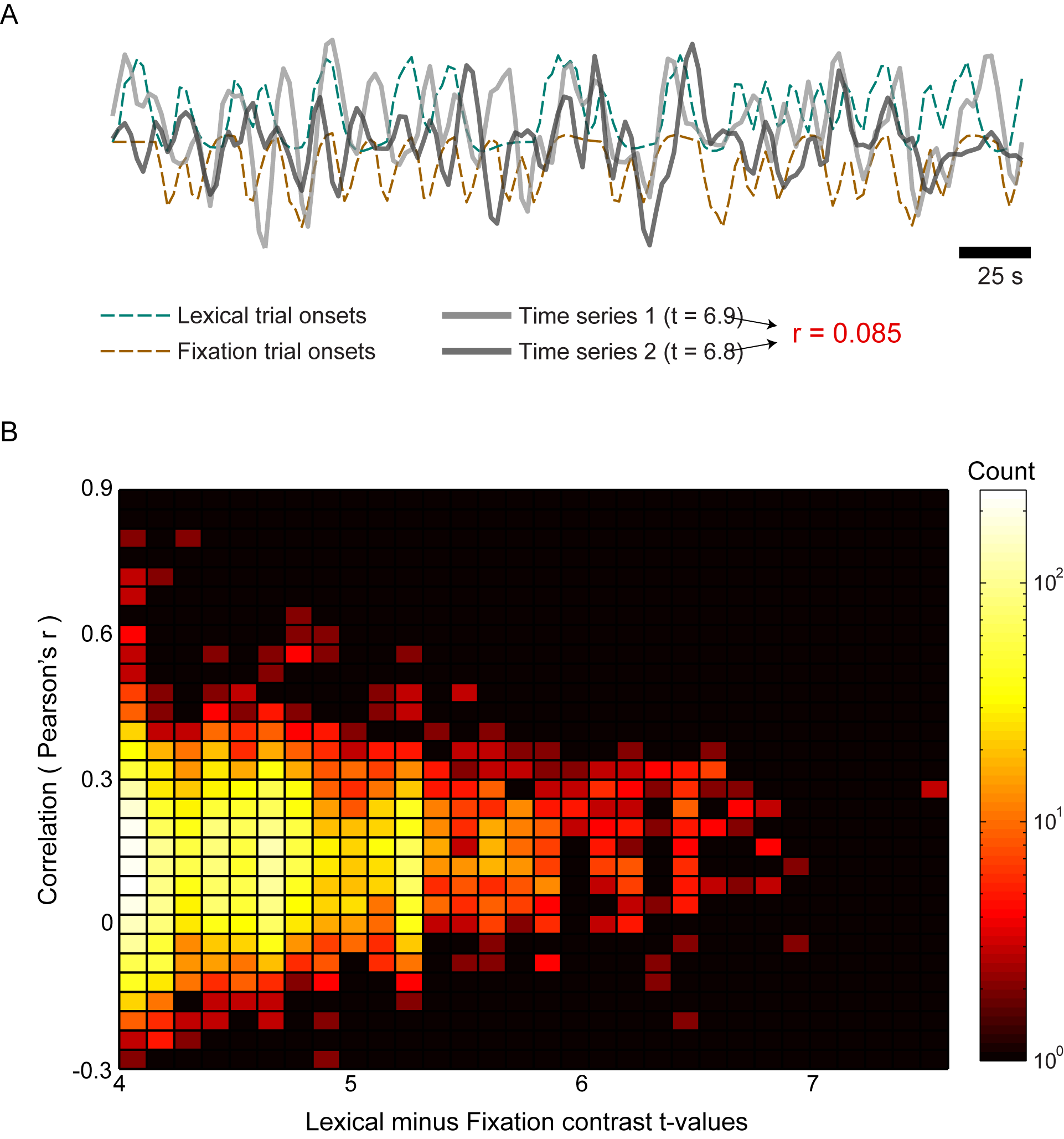

Supplement: Figure S1 — High activation levels are not related to high time-series correlation. (A) Two example time series of activation from two separate ROIs (light gray and dark gray solid lines) are superimposed on stimulus onsets for lexical (dashed green line) and fixation (dashed orange line) trials, convolved with a canonical hemodynamic response. The fixation trial stimulus onsets are inverted (onset corresponds with downward inflection) for visual clarity. Both ROIs exhibit high activation levels (t>6.5 for lexical minus fixation contrast), yet have very low correlation between them (Pearson’s product moment r = 0.085). (B) Time series from all nodes with high activation (t>4) are binned according to activation level, and all time series in the same bin are cross-correlated. The resultant correlations are binned along the y-axis to yield a 2-D histogram of how correlations are distributed for every t-value bin. (TIF) [file pone.0059204.s001.tif]

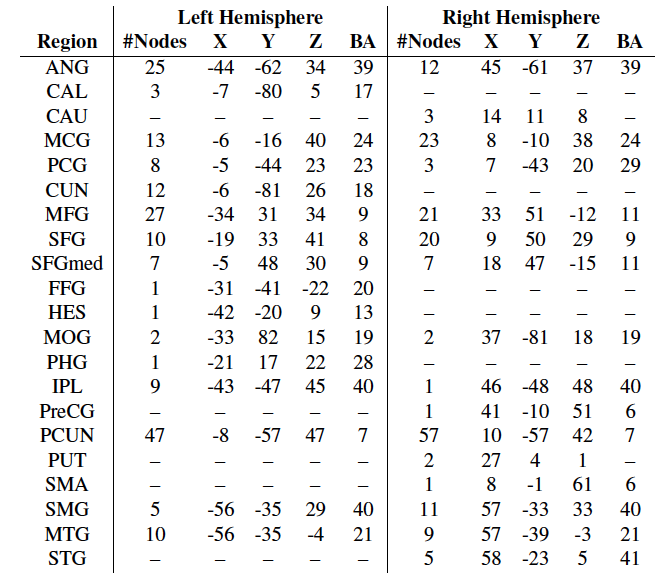

Supplement: Table S2 — Details of anatomical regions in the task-negative areas. Columns refer to the number of nodes within each anatomical region (as defined by the AAL atlas), spatial coordinates of the center of mass, and Brodmann Area (BA). X, Y, and Z refer to MNI coordinates. Region names: ANG: Angular Gyrus, CAL: Calcarine, CAU: Caudate, MCG: Middle Cingulate, PCG: Posterior Cingulate, CUN: Cuneus, MFG: Middle Frontal Gyrus, SFG: Superior Frontal Gyrus, SFGmed: Medial Superior Frontal Gyrus, FFG: Fusiform, HES: Heschel’s Gyrus, MOG: Middle Occipital Gyrus, PHG: Parahippocampal Gyrus, IPL: Inferior Parietal Lobule, PreCG: Precentral Gyrus, PCUN: Precuneus, PUT: Putamen, SMA: Superior Motor Area, SMG: Supramarginal Gyrus, MTG: Middle Temporal Gyrus, STG: Superior Temporal Gyrus. (DOCX) [file pone.0059204.s003.docx]
