# Supplementary material for: Changes in Task-Related Functional Connectivity across Multiple Spatial Scales Are Related to Reading Performance
Source: PLoS One. 2013 Mar 27;8(3):e59204. doi: 10.1371/journal.pone.0059204 (PMC3609744; doi:10.1371/journal.pone.0059204)
Supplement: Supplementary file 2 [file pone.0059204.s002.docx]

| Region | Left Hemisphere | | | | | Right Hemisphere | | | | |
|---|---|---|---|---|---|---|---|---|---|---|
| | #Nodes | X | Y | Z | BA | #Nodes | X | Y | Z | BA |
| CAL | 20 | -7 | -80 | 5 | 19 | 16 | 16 | -74 | 8 | 23 |
| MCG | 4 | -6 | -16 | 40 | 24 | 2 | 8 | -10 | 38 | 24 |
| IFGoperc | 17 | -49 | 11 | 18 | 44 | – | – | – | – | – |
| ORBinf | 4 | -36 | 29 | -13 | 47 | – | – | – | – | – |
| IFGtriang | 14 | -46 | 29 | 13 | 45 | – | – | – | – | – |
| SFG | 1 | -19 | 33 | 41 | 8 | – | – | – | – | – |
| SFGmed | 11 | -5 | 48 | 30 | 9 | 2 | 18 | 47 | -15 | 11 |
| FFG | 18 | -31 | -41 | -22 | 37 | 4 | 34 | -40 | -22 | 37 |
| HIP | 1 | -25 | -22 | -11 | 28 | – | – | – | – | – |
| INS | 14 | -35 | 5 | 2 | – | 4 | 39 | 5 | 1 | – |
| LING | 13 | -15 | -69 | -6 | 18 | 12 | 16 | -68 | -5 | 18 |
| IOG | 15 | -36 | -80 | -9 | 19 | 2 | 38 | -83 | -9 | 19 |
| MOG | 11 | -33 | -82 | 15 | 19 | 2 | 37 | -81 | 18 | 19 |
| SOG | 3 | -17 | -86 | 27 | 18 | – | – | – | – | – |
| IPL | 2 | -43 | -47 | 45 | 40 | – | – | – | – | – |
| SPG | 1 | -24 | -61 | 58 | 7 | – | – | – | – | – |
| PoCG | 2 | -43 | -24 | 47 | 3 | – | – | – | – | – |
| PreCG | 20 | -39 | -7 | 50 | 6 | – | – | – | – | – |
| PCUN | 1 | -8 | -57 | 47 | 7 | – | – | – | – | – |
| SMA | 19 | -6 | 4 | 60 | 6 | 5 | 8 | -1 | 61 | 6 |
| ITG | 10 | -50 | -29 | -25 | 20 | 3 | 53 | -32 | -24 | 20 |
| MTG | 6 | -56 | -35 | -4 | 21 | – | – | – | – | – |
| THA | 3 | -11 | -19 | 7 | – | 12 | 13 | -19 | 7 | – |
